# Compliance with Once-Daily versus Twice or Thrice-Daily Administration of Antibiotic Regimens: A Meta-Analysis of Randomized Controlled Trials

**DOI:** 10.1371/journal.pone.0116207

**Published:** 2015-01-05

**Authors:** Matthew E. Falagas, Apostolos K. A. Karagiannis, Theodora Nakouti, Giannoula S. Tansarli

**Affiliations:** 1 Alfa Institute of Biomedical Sciences (AIBS), Athens, Greece; 2 Department of Internal Medicine—Infectious Diseases, Iaso General Hospital, Iaso Group, Athens, Greece; 3 Department of Medicine, Tufts University School of Medicine, Boston, Massachusetts, United States of America

## Abstract

**Objective:**

To investigate whether compliance of patients to antibiotic treatment is better when antibiotics are administered once than multiple times daily.

**Methods:**

We performed a systematic search in PubMed and Scopus databases. Only randomized controlled trials were considered eligible for inclusion. Compliance to antibiotic treatment was the outcome of the meta-analysis.

**Results:**

Twenty-six studies including 8246 patients with upper respiratory tract infections in the vast majority met the inclusion criteria. In total, higher compliance was found among patients treated with once-daily treatment than those receiving treatment twice, thrice or four times daily [5011 patients, RR=1.22 (95% CI, 1.11, 1.34]. Adults receiving an antibiotic once-daily were more compliant than those receiving the same antibiotic multiple times daily [380 patients, RR=1.09 (95% CI, 1.02, 1.16)]. Likewise, children that received an antibiotic twice-daily were more compliant than those receiving the same antibiotic thrice-daily [2118 patients, RR=1.10 (95% CI, 1.02, 1.19)]. Higher compliance was also found among patients receiving an antibiotic once compared to those receiving an antibiotic of different class thrice or four times daily [395 patients, RR=1.20 (95% CI, 1.12, 1.28)]. The finding of better compliance with lower frequency daily was consistent regardless of the study design, and treatment duration.

**Conclusion:**

This meta-analysis showed that compliance to antibiotic treatment might be associated with higher when an antibiotic is administered once than multiple times daily for the treatment of specific infections and for specific classes of antibiotics.

## INTRODUCTION

Infections are commonly encountered diseases in every day clinical practice and affect both people with co-morbidities and people that are otherwise healthy. Timely administration of the appropriate empirical antibiotic treatment has been associated with survival in patients with severe infections.[1–4] However, patients’ compliance is another important parameter that may also account for the response to treatment in less severe infections where the antibiotic treatment is administered *per os*. The frequency of the daily dosing is among others a factor affecting compliance to treatment.[5]

Controversial results have been published so far regarding compliance to antibiotic treatment showing that once-daily regimens lead to better compliance than the regimens administered multiple times daily[6,7] or vice versa,[8] or even that there is no difference in compliance between antibiotic regimens administered once and multiple times daily.[9,10]

In this context, we aimed to systematically review and synthesize the available evidence with the methodology of meta-analysis in order to determine whether the administration of once-daily regimens results in higher compliance to treatment than regimens administered multiple times daily.

## METHODS

### Literature search

We systematically reviewed the literature in the PubMed and Scopus databases up to June 2013. The following search term was used without a limit in the year of publication: “(once daily OR twice daily OR three times daily OR thrice daily OR four times daily) AND (antibiotic OR antimicrobial OR anti infective) AND (compliance OR adherence)”. Furthermore, the bibliographies of all relevant articles were hand-searched in order to retrieve additional potentially eligible studies. Articles published in a language other than English, German, French, Spanish, Italian, or Greek were not evaluated.

### Study selection

Any randomized controlled trial (RCT) that compared the compliance between patients receiving antibiotic treatment once-daily and those receiving the treatment multiple times (twice, thrice, or four times) daily were considered eligible for inclusion in the review. Studies comparing the compliance between patients treated with twice-daily antibiotic regimens with those treated with thrice or four times daily regimens were also included. Non-randomized studies were excluded.

### Data extraction

The extracted data included the main characteristics of each study (first author’s name, year of publication, study design and period country), number of included patients, age group of patients (adults or children), site of infection, and detailed description of the antibiotic treatment regimens that were administered (medication, amount of dose, frequency of administration, treatment duration). Finally, the definition of compliance that was used in each study was recorded.

### Definitions and outcomes

The outcome of the meta-analysis was compliance to antibiotic treatment defined according to the definitions used by the authors of the included studies. Three levels of analysis were developed according to the compared antibiotics. The primary analysis reports on comparison between dosing regimens of the same antibiotic or between regimens of antibiotics of the same class (i.e. between penicillins or between cephalosporins). The secondary analysis included studies comparing dosing regimens of antibiotics of a same broader class of antibiotics (i.e. between beta-lactams). Finally, the third analysis reports on comparison between dosing regimens of antibiotics of different classes (i.e between a beta-lactam and a macrolide). In each of the aforementioned analyses, subgroup analysis according to age group was performed.

### Statistical analysis

The meta-analysis was performed with Review Manager for Windows, version 5.1. Pooled risk ratios (RR) and 95% confidence intervals (CI) were calculated. Statistical heterogeneity among studies was assessed by using a *χ* ^2^ test (P < .10 was defined to indicate significant heterogeneity) and *I* ^2^ (assessed the degree of heterogeneity). The Mantel-Haenszel fixed effect model (FEM) was used when there was no significant statistical heterogeneity between the studies; otherwise, the random effects model (REM) was used as appropriate.

## RESULTS

**Figure 1:**
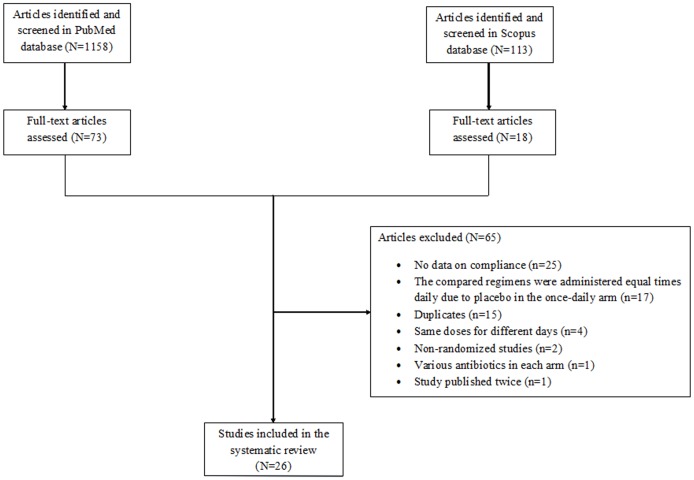
Flow diagram of the systematic search and study selection process.

A total of 1271 articles (1158 articles from PubMed and 113 articles from Scopus) were retrieved during the search process 26 out of which were finally included in the review.[6–31] Seventeen double-blind studies were excluded because the compared regimens were administered equal times daily due to placebo in the once-daily arm.[32–48] One study was excluded because various antibiotics were administered in each arm.[49] Seventeen out of 26 RCTs were open–label,[7–9,11,12,14,15,18,19,21,22,24,26–30] while the remaining nine studies were single-blind.[6,10,13,16,17,20,23,25,31] The detailed study selection process is depicted in Fig. 1. The characteristics of the included studies are presented in Table 1 and Table 2.

**Table 1:** Characteristics and outcomes of the studies comparing compliance with once versus twice/thrice/four times daily antibiotic regimens.

**First author, Year**	**Study design; study period, country**	**Number of pts; age group, site of infection**	**Antibiotic regimens**	**Compliance**		**Definition of compliance**
				**OD (%)**	**BID/TID/QID (%)**	
Hosie, 1995[25]	MC single-blind; 1991–1992, UK	212; >18yo, AECB	Dirithromycin 500 mg OD for 5 d vs clarithromycin 250 mg BID for 7 d	100/104 (96.2)	96/108 (88.9)	Took all the medication
Kardas, 2007[7]	SC open-label; NR, Poland	119; >18yo, respiratory tract infections	Clarithromycin in modified release formulation 500 mg OD vs clarithromycin 250 mg BID for 7 d	54/58 (93.1)	50/61 (82)	Ratio of the number of container openings to the number of prescribed doses
Martinot, 2001[6]	SC single-blind; NR, Belgium	250; >35yo, AECB	Clarithromycin MR 500 mg OD vs amoxicillin/clavulanic acid 500 mg/125 mg TID for 7 d	121/127 (95.3)	98/123 (79.7)	100% compliance
Lennon, 2008[9]	SC open-label; 1996–1998, New Zealand	353; 5–12 yo, GABHS pharyngitis	Oral amoxicillin 1500 mg OD (or 750 mg if bodyweight was <30 kg) vs oral penicillin V 500 mg BID (or 250 mg if bodyweight was >20 kg) for 10 d	176/177 (99.4)	175/176 (99.4)	Received >80% of the scheduled doses
Adam, 2001[11]	MC open-label; 1995–1998, Germany	2099; children, upper respiratory tract infections	Ceftibuten 9 mg/kg OD for 5 d vs penicillin V 50,000 I.E./kg TID for 10 d	492/507 (97)	1305/1412 (92.4)	NR
García Callejo, 1998[21]	SC open-label; NR, Spain	145; 3–18yo, ENT infections	Azithromycin 10 mg/kg in children or 500 mg/day in adults OD for 3 d vs amoxicillin/clavulanic acid 40 mg/kg in children or 500 mg/kg in adults TID or cefaclor 40 mg/kg in children or 250 mg/kg in adults TID for 7–14 d	67/67 (100)	65/78 (83.3)	NR
Clegg, 2006[10]	SC single-blind; 2001–2003, USA	590;3–18 yo, GAS pharyngitis	<40kg: Amoxicillin OD 750 mg vs amoxicillin BID 375 mg for 10 d / >40kg: Amoxicillin OD 1000 mg vs amoxicillin BID 500 mg for 10 d	271/294 (92.2)	270/296 (91.2)	Daily logs returned at visit 2
Ballantyne, 1985[12]	SC open-label; NR, NR	200; 6–80yo, skin and soft-tissue infections	Cefadroxil 1000 mg OD vs cefaclor 250 mg TID for 10 d	98/100 (98)	23/100 (23)	Took all the medication
Linder 1993[26]	MC open-label; NR, USA	289; 1–18yo, skin infections	Cefadroxil 30 mg/kg or caps 500 mg OD for 10 d vs cephalexin 30 mg/kg/day or caps 500 mg/d BID for 10 d	148/156 (94.9)	85/133 (63.9)	Took all the medication
Gooch, 1997[24]	MC open-label, randomized; NR, USA	286; 6m-12yo, acute otitis media	Cefixime 8 mg/kg OD vs amoxicillin/clavulanic acid 40 mg/kg/day TID for 10 d	147/148 (99.3)	82/138 (59.4)	Convenient administration schedule
Edelstein, 1993[19]	MC open-label; NR, USA	103; >18yo, sinusitis	Cefixime 400 mg OD vs amoxicillin 500 mg TID for 10 d (or if needed 4 d more)	51/55 (92.7)	34/48 (70.8)	Less than the prescribed doses
Owen,1993[28]	SC open-label; 1987–1988, USA	152; 2 mo-6yo, acute otitis media	Cefixime 8 mg/kg/d OD vs amoxicillin 40 mg/kg/d TID for 10 d	77/80 (96.3)	61/72 (84.7)	≥80% of the prescribed medication
Venuta, 1998[31]	SC single-blind; 1994–1997, Italy	164; 4–13yo, streptococcal pharingitis	Azithromycin 10 mg/kg OD for 3 d vs clarithromycin 7.5 mg/kg BID for 10 d	76/81(93.8)	64/83 (77.1)	Compliance with the allocated treatment
Disney, 1990[18]	MC open-label; 1989, USA	180; >2yo, GABHS tonsillopharyngitis	Cefadroxil 30 mg/kg OD vs erythromycin 30 mg/kg QID for 10 d	92/96 (95.8)	77/84 (91.7)	Amount of medication returned
Mita,2003[27]	MC open-label; 2002–2003, Japan	49; >60yo, respiratory tract infections	Levofloxacin 300 mg OD vs levofloxacin 100 mg TID for 7 d	22/25 (88)	21/24 (87.5)	NR

**Abbreviations:** MC: multicenter, SC: single-center, RCT: randomized controlled trial, NR: not reported, yo: years old, mo: months, d: days, OD: once-daily, BID: twice-daily, TID: thrice-daily, QID: four times daily, AECB: acute exacerbation of chronic bronchitis, GABHS: group A b-haemolytic streptococcal, GAS: group A streptococcal, ENT: ear, nose and throat infections

**Table 2:** Characteristics and outcomes of the studies comparing compliance with twice versus /thrice/four times daily antibiotic regimens.

**First author, Year**	**Study design; study period, country**	**Number of pts; aging group, site of infection**	**Antibiotic regimens**	**Compliance**		**Definition of compliance**
				**BID (%)**	**TID/QID (%)**	
Cheung, 1988[14]	SC open-label; NR, UK	77; >50yo, urinary tract infections	Trimethoprim 2 tb of 100 mg BID vs cefalexin 250 mg QID for 7 d	31/44 (70.5)	13/33 (39.4)	28 and 14 pill box openings respectively
Cohen, 1996[15]	MC open-label; 1993–1995, France	312; 3–15yo, GAS tonsillopharyngitis	Amoxicillin 50 mg/kg/day BID for 6 d vs penicillin V 45 mg/kg/day TID for 10 d	139/159 (87.4)	103/153 (67.3)	12 doses of amoxicillin and 30 doses of penicillin V according to the diary cards
Behre, 1997[13]	MC single-blind; NR, Europe	463; 2–12yo, acute otitis media	Amoxicillin/clavulanic acid 70/10/mg/kg/d BID vs amoxicillin/clavulanic acid 60/15/mg/kg/d TID for 10 d	192/231 (83.1)	169/232 (72.8)	80% of the volume of medication over a 7–10 d of therapy
Pichichero,1999[29]	SC open-label; 1995–1997, USA	478; 2–19yo, GABHS tonsillopharyngitis	Penicillin V 500 mg BID vs penicillin V 250 mg TID for 10 d	215/239 (90)	208/239 (87)	Positive urine test at 5 day of treatment
Gooch, 1993[23]	MC single-blind; 1989–1990, USA	484; 2–13yo, GABHS pharyngitis	Cefuroxime axetil suspension 20 mg/kg/d BID vs penicillin V suspension 50 mg/kg/d TID for 10 d	300/314 (95.5)	157/170 (92.4)	Antibiotic present in urine bioassay
Richard, 1981[30]	MC open-label; NR, USA	146; women 17–37yo, urinary tract infections	Bacampicillin 400 mg BID vs amoxicillin 250 mg TID for 10 d	79/83 (95.2)	78/82 (95.1)	Comply with the dosage regimen
Damrikarnlert, 2000[17]	MC single-blind; 1996–1998, South America, Asia, Africa	415; 2m-12yo, acute otitis media	Amoxicillin/clavulanic acid 45/6.4 mg/kg/day BID vs amoxicillin/clavulanic acid 40/10 mg/kg/day TID for 7 or 10 d	173/209 (82.8)	151/206 (73.3)	At least 80% of the suspension at 7–10 d treatment
Gerber, 1985[8]	SC open-label; 1983–1984, USA	97; 2–16yo, GABHS pharyngitits	Penicillin V 250 mg BID vs penicillin V 250 mg TID for 10 d	41/48 (85.4)	46/49 (93.9)	Antibiotic activity in urine
Fyllingen, 1991[20]	SC single-blind; 1987–1990, Norway	131; >6m, upper respiratory tract infections	Phenoxymethilpenicillin same total dose BID vs TID (1/2–1yo 500 mg/d, 1–5yo 1000 mg/d, 5–12yo 1320 or 1980 mg/d and >12yo 3960 or 2640 mg/d for 5 or 7 d)	70/71 (98.6)	58/60 (96.7)	80% of the medication
Gehanno, 1994[22]	MC open-label; 1990–1991, France, Finland	260; 3m-11yo, acute otitis media	Cefpodoxime proxetile 8 mg/kg/d BID vs amoxicillin/clavulanic acid 40/10mg/kg/d TID for 8 d	131/131 (100)	126/129 (97.7)	80% of the scheduled doses or receiving <5 d treatment at the prescribed dose
Cook, 1996[16]	MC single-blind; NR, UK	353; 2–12yo, lower respiratory tract infections	Amoxicillin/clavulanic acid 25/3.6 mg/kg/d BID vs amoxicillin/clavulanic acid 20/5mg/kg/d TID for 7 d	164/182 (90.1)	137/171 (80.1)	80% compliance

**Abbreviations:** MC: multicenter, SC: single-center, RCT: randomized controlled trial, NR: not reported, yo: years old, mo: months, d: days, OD: once-daily, BID: twice-daily, TID: thrice-daily, QID: four times daily, AECB: acute exacerbation of chronic bronchitis, GABHS: group A b-haemolytic streptococcal, GAS: group A streptococcal, ENT: ear, nose and throat infections

The antibiotics most commonly administered were penicillins (18 studies),[6,8–11,13,15–17,19–24,28–30] followed by cephalosporins (10 studies)[11,12,14,18,19,22–24,26,28] and macrolides (6 studies).[6,7,18,21,25,31] Levofloxacin[27] and trimethoprime-sulfamethoxazole[14] were used each one in one trial. Amoxicillin/clavulanic acid was the most commonly administered penicillin.[6,13,16,17,21,22,24] Twenty-two studies referred to respiratory tract infections (8 tonsillopharyngitis,[8–10,15,18,23,29,31] 5 acute otitis media,[13,17,22,24,28] 3 lower respiratory tract infections,[6,16,25] 1 sinusitis,[19] and 5 undetermined respiratory tract infections[7,11,20,21,27]), two studies to urinary tract infections[14,30] and the remaining 2 to skin infections.[12,26]

**Figure 2:**
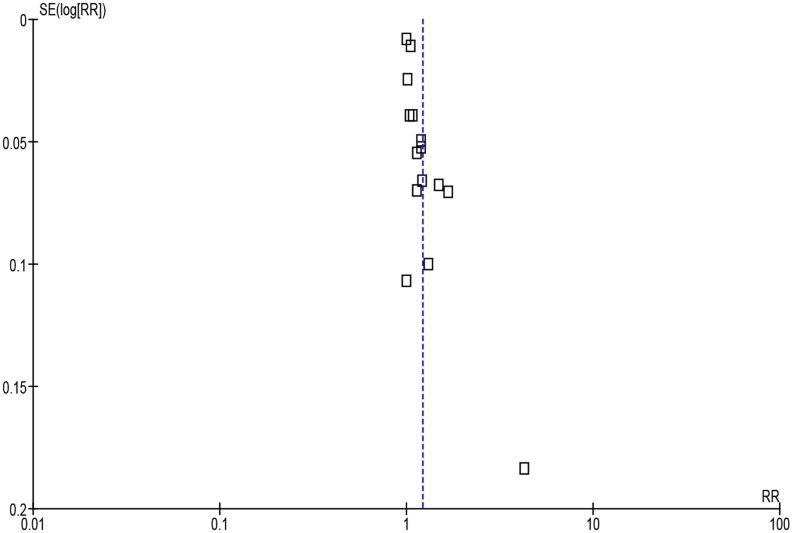
Forest plot depicting the risk ratios (RR) of compliance of patients receiving antibiotic treatment once-daily versus multiple times daily. *(Vertical line = “no difference” point between the two regimens. Squares = risk ratios; Diamonds = pooled risk ratios for all studies. Horizontal lines = 95% CI)*.

**Figure 3:**
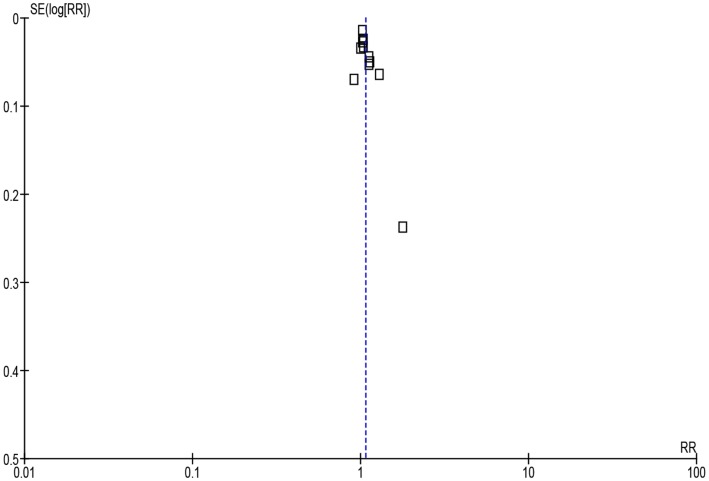
Forest plot depicting the risk ratios (RR) of compliance of patients receiving antibiotic treatment twice-daily versus thrice or four times daily.

Pooling all the studies showed that compliance was significantly higher with once versus multiple times daily regimens [Fig. 2, 5011 patients, RR = 1.22 (95% CI, 1.11, 1.34)] and with twice versus thrice or four times daily regimens [Fig. 3, 3235 patients, RR = 1.07 (95% CI, 1.01, 1.13)]. Considerable heterogeneity was detected in both analyses (*I* ^2^ = 97% and 82%, respectively).

### Same antibiotic or antibiotics of the same class compared

**Figure 4:**
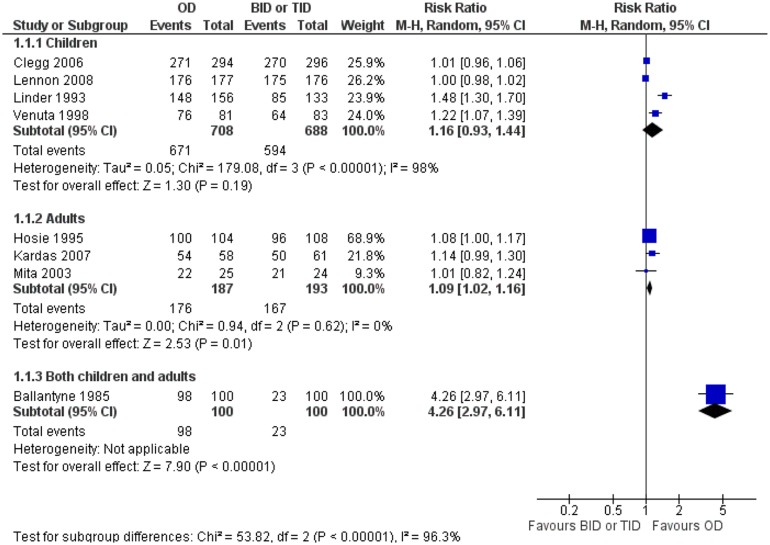
Forest plot depicting the risk ratios (RR) of compliance of patients receiving an antibiotic once-daily versus the same antibiotic or antibiotic of the same class twice or thrice-daily.

**Figure 5:**
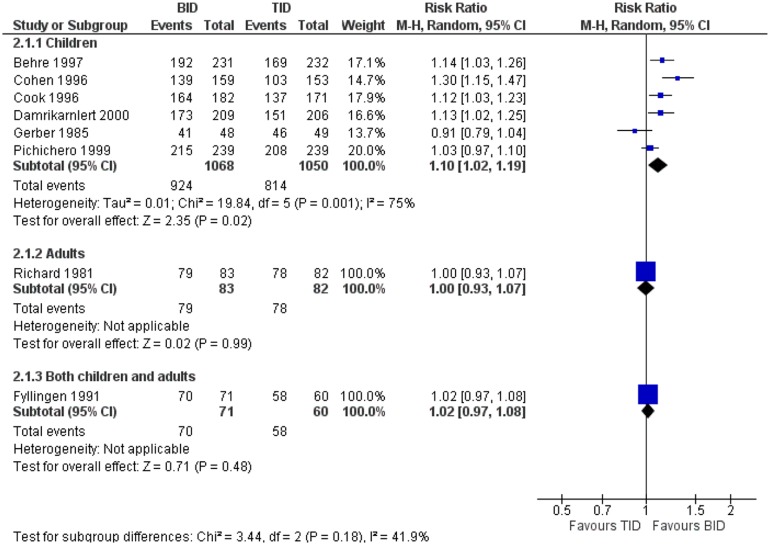
Forest plot depicting the risk ratios (RR) of compliance of patients receiving an antibiotic twice-daily versus the same antibiotic or antibiotic of the same class thrice-daily.

Adults receiving once-daily regimens were more compliant than adults receiving twice or four times daily regimens [380 patients, RR = 1.09 (95% CI, 1.02, 1.16)]. No heterogeneity was detected in this analysis (*I* ^2^ = 0%). On the other hand, no significant difference in compliance between the compared regimens was found in the child population [Fig. 4, 1396 patients, RR = 1.16 (95% CI, 0.93, 1.44)]. Considerable heterogeneity was detected in this analysis, as well (*I* ^2^ = 98%). Higher compliance was found in one study including both adults and children with once versus thrice-daily regimens [patients, RR = 4.26 (95% CI, 2.97, 6.11)].[12] Also, higher compliance was observed among adults receiving twice-daily regimens compared to those receiving thrice-daily regimens [Fig. 5, 2118 patients, RR = 1.10 (95% CI, 1.02, 1.19)]. Statistical heterogeneity was considerable in this analysis (*I* ^2^ = 75%). One study reported on children and another one on both adults and children.[20,30] No significant difference in compliance between twice and thrice-daily regimens was observed in any of these studies, [165 patients, RR = 1.00 (95% CI, 0.93, 1.07)] and [131 patients, RR = 1.02 (95% CI, 0.97, 1.08)] respectively.

### Antibiotics of the same broad class compared

**Figure 6:**
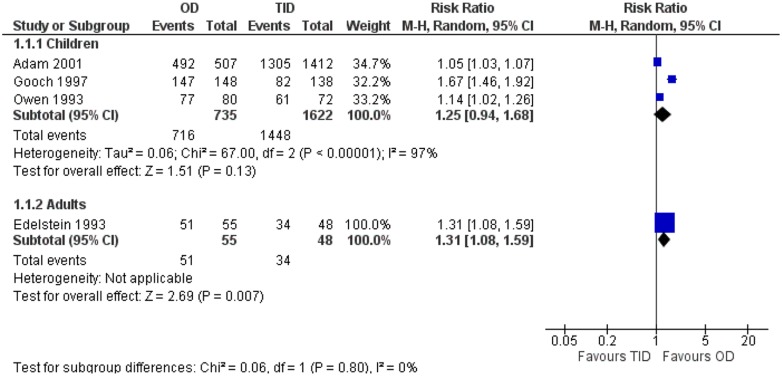
Forest plot depicting the risk ratios (RR) of compliance of patients receiving an antibiotic once-daily versus an antibiotic of the same broad class thrice-daily.

No significant difference in compliance was found between children receiving once-daily regimen and those receiving thrice-daily regimen [Fig. 6, 2357 patients, RR = 1.25 (95% CI, 0.94, 1.68)]. Considerable heterogeneity was detected in this analysis (*I* ^2^ = 97%). One study reporting on adults showed higher compliance with once versus thrice-daily regimens [103 patients, RR = 1.31 (95% CI, 1.08, 1.59)]. The compliance was not different nor between children receiving twice and those receiving thrice-daily regimens [744 patients, RR = 1.03 (95% CI, 1.00, 1.06)]. No heterogeneity was detected in this analysis (*I* ^2^ = 0%).

### Antibiotics of different classes compared

**Figure 7:**
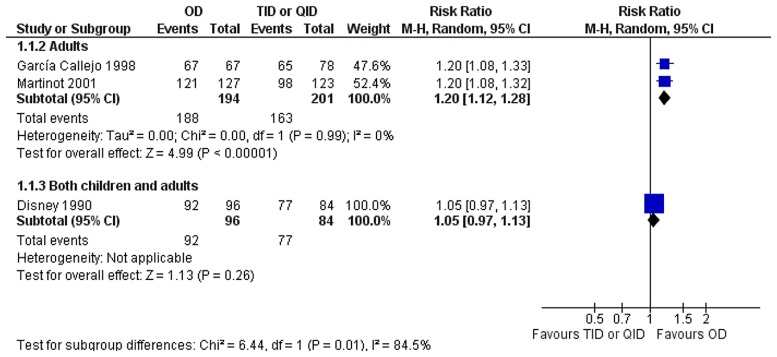
Forest plot depicting the risk ratios (RR) of compliance of patients receiving an antibiotic once-daily versus an antibiotic of different class thrice or four times daily.

The compliance was higher among adults treated with once-daily regimen than those treated with thrice or four times daily regimens [Fig. 7, 395 patients, RR = 1.20 (95% CI, 1.12, 1.28)]. No heterogeneity was detected in this analysis (*I* ^2^ = 0%). One study reporting on both adults and children did not show any difference in compliance between the compared groups [180 patients, RR = 1.05 (95 CI, 0.97, 1.13)].[18]

**Study design: open-label versus single-blind.** Analyzing the compliance regarding the study design of the included studies, it was shown that the compliance was significantly higher when an antibiotic was administered once than multiple times daily, both in open-label and in single-blind RCTs, [3795 patients, RR = 1.28 (95% CI, 1.12, 1.46)] and [1216 patients, RR = 1.11 (95% CI, 1.01, 1.22)] respectively. Considerable heterogeneity was detected in both analyses (*I* ^2^ = 98% and 80%, respectively).

**Definition of compliance: received all versus high amount of the medication.** The analysis regarding the definition of compliance showed that when as compliant patients were defined those who received all the medication, there was higher compliance in patients receiving the drug once-daily than those receiving the drug multiple times daily [1054 patients, RR = 1.54 (95% CI, 1.14, 2.09]. Contrariwise, when as compliant patients were defined those who received high amount but not all the medication, no significant differences were found between the once-daily and multiple times daily groups [505 patients, RR = 1.06 (95% CI, 0.82, 1.38)]. Considerable heterogeneity was detected in both analyses (*I* ^2^ = 96%).

**Duration of treatment: ≤ 7 versus > 7 days.** When analysis was performed according to the duration of the antibiotic treatment, the compliance was higher in the once-daily group compared to the multiple times daily group, both in studies with short duration of treatment (≤ 7 days) [630 patients, RR = 1.12 (95% CI, 1.05, 1.19)] and in those with longer duration (> 7 days) [1867 patients RR = 1.34 (95% CI, 1.03, 1.74)]. No heterogeneity was detected in the former analysis (*I* ^2^ = 19%), while considerable in the latter analysis (*I* ^2^ = 99%).

## DISCUSSION

This meta-analysis revealed that patients who received antibiotic treatment once-daily had higher compliance than those who received antibiotic treatment multiple times daily. Of interest, this finding was observed both in open-label and in single-blind RCTs.

Previous studies have suggested that the clinical effectiveness with once-daily regimens may be non-inferior to multiple daily dosing regimens.[50–52] Regarding the same comparison, this meta-analysis showed that compliance to treatment appears to be higher with once than multiple daily dosing regimens. In particular, higher compliance in the once-daily group compared to the multiple times daily group was observed both for those who received treatment for ≤ 7 days and for those who received treatment for > 7 days. The analysis regarding the definition of compliance used among the included studies showed that the compliance was higher with the once-daily regimen than the regimen administered multiple times daily only in studies where a patient was considered compliant when he took all the doses of the medication during treatment. Analyses comparing compliance according to the type of antibiotic administered in each arm (i.e. same antibiotic, same class of antibiotics, different broad classes of antibiotics) were also performed in this meta-analysis. In particular, adults receiving antibiotic treatment once-daily had higher compliance than those receiving the same antibiotic or antibiotic of the same class twice or thrice-daily, while compliance was higher in children receiving antibiotic treatment twice-daily compared to those receiving the same antibiotic or antibiotic of the same class thrice-daily. When antibiotics of different broad classes were compared, the compliance was higher in adults who received antibiotic treatment once-daily than those who received another antibiotic thrice or four times daily. Most studies showed individually numerical superiority of the regimen administered fewer times daily than that administered more times daily, or no difference between the compared regimens.

Expectedly, the more times daily patients took a medication, the less compliant were. In fact, the lowest percentages of compliance were observed among patients treated with thrice-daily regimens or four times daily regimen in one study.[8] The most rational reason that patients were not as compliant with multiple times daily as with once-daily regimens is the possibility of forgetting to take a dose when a regimen is administered over once-daily. Besides, some patients may underestimate the omission of a dose, and thus may be less compliant when they must receive an antibiotic multiple times daily. Indeed, multiple times of daily administration of a drug and long-duration treatment make the compliance difficult and lead to poor treatment.[5,53,54] As far as children are concerned, one may expect that compliance in this population may not differ according to the number of times daily that a regimen is administered, since taking the medication is not at their discretion but instead, guardians are responsible for it. Actually, no difference was found between fewer and more times daily in most analyses except that on the same antibiotic or antibiotic of the same class between the two arms given twice versus thrice-daily. Specifically, children who received penicillins twice-daily had higher compliance than the children who received penicillins thrice-daily. This could partially be justified by the fact that as the number of daily doses increases, the possibility the guardian forgets to give the medication increases, as well.

High compliance may lead to clinical success but low compliance may result in treatment failure, emergence of resistant strains, and increased healthcare costs through relapses of infection and hospitalizations.[54–57] Apart from the frequency of the daily dosing, other factors can also affect compliance to treatment.[5] These factors can be categorized as patient-centered (i.e. age, gender, health literacy), therapy-related (i.e. taste or odor of the medication, adverse events, long duration of treatment), as well as factors associated with the healthcare system, social and economic status of the patient, and the severity of disease.[5] Last, another interesting view is that patients may obtain the highest compliance around doctor’s visits[58,59] which means that contact between patient and doctor during treatment may result in higher compliance. It is now evident that clinicians should take into consideration the patient’s compliance before prescribing an antibiotic.

To our knowledge this is the first meta-analysis study focusing on the compliance to antibiotic treatment according to the number of doses per day. The finding of the meta-analysis is consistent with a previous review that studied the association between dose regimens and medication compliance.[53] In that review, the authors pinpointed the value of simplicity showing that less frequent dosing regimens lead to higher compliance across a variety of drugs. Same results have been reported in several previous studies demonstrating better compliance with lower frequency daily dosing in various medications, such as antihypertensive,[60,61] antiviral,[62,63] inhaled drugs,[64,65] or even eye drops[66,67] and anti-acne drugs.[68]

Our findings should be interpreted in view of important limitations. First, considerable statistical heterogeneity was detected in most analyses. In addition, it should be emphasized that compliance was not included among the primary outcomes in none but one included study.[7] Furthermore, all patients were from RCTs, while it has been suggested that patients included in RCTs may be different from those viewed in clinical practice[69] and this may have contributed in an overall high compliance in both treatment arms. Still, the included infections were not severe and the antibiotics were administered for a short period of time which also may lead to a high overall compliance to treatment. This is juxtaposed to severe infections such as tuberculosis for which patients receive long-duration treatment and have low compliance.[70] The definition of compliance which differed among the included studies as well as the method of assessment of compliance is an additional limitation that should be taken into account in the evaluation of our findings. Finally, the included studies were from different countries and continents and it has been suggested that compliance may be higher among white patients compared to African American, Hispanics or Asian.[71,72]

In conclusion, considering the limitations surrounding this meta-analysis once-daily antibiotic treatment might be associated with higher compliance than treatment administered multiple times daily in specific populations, for specific sites of infections and specific classes of antibiotics. Since higher compliance to treatment may imply higher clinical effectiveness, the frequency of the dosing schedule of an antibiotic is an additional parameter that could be considered before prescription.

## Supporting Information

S1 PRISMA Checklist PRISMA Checklist. (DOC)
